# Current Status and Health Risk Assessment of Heavy Metals Contamination in Tea across China

**DOI:** 10.3390/toxics11080662

**Published:** 2023-08-01

**Authors:** Chenglin Hu, Xiuying Zhang, Nan Zhan, Youcun Liu

**Affiliations:** 1 International Institute for Earth System Science, Nanjing University, Nanjing 210023, China; mg21270067@smail.nju.edu.cn (C.H.);; 2 Key Laboratory of Urban Land Resources Monitoring and Simulation, Ministry of Natural Resources, Shenzhen 518000, China; 3 Jiangsu Center for Collaborative Innovation in Geographical Information Resource Development and Application, Nanjing 210023, China; 4 School of Geography and Tourism, Jiaying University, Meizhou 514015, China

**Keywords:** tea plantation soils, drinking tea, exposure, non-carcinogenic, carcinogenic

## Abstract

Tea is a non-alcoholic beverage popular among Chinese people. However, due to the application of chemical and organic fertilizers in the tea planting process, the environment pollutionaround the tea plantation, and the instruments used in the processing, heavy metal elements will accumulate in the tea, which brings health risks for tea consumers. This study summarized heavy metal concentrations from 227 published papers and investigated the current contamination status of tea and tea plantation soils, and, finally, the risk of heavy metal exposure to tea consumers in China is assessed, in terms of both non-carcinogenic and carcinogenic risk. The average contamination of six heavy metals in tea—arsenic (As), cadmium (Cd), chromium (Cr), copper (Cu), mercury (Hg), and lead (Pb)—were 0.21, 0.14, 1.17, 14.6, 0.04, and 1.09 mg/kg, respectively. The areas with high concentrations of heavy metals in tea were concentrated primarily in southwest China, some areas in eastern China, and Shaanxi Province in northwest China. The non-carcinogenic risks of heavy metals in tea are all within safe limits. The national average HI value was 0.04, with the highest HI value of 0.18 in Tibet, which has the largest tea consumption in China. However, the carcinogenic risks of Cd in Shaanxi Province, Anhui Province, and southwest China exceed the acceptable range, and due attention should be given to these areas.

## 1. Introduction

China was the first country in the world to discover, utilize, and cultivate tea trees. Tea contains hundreds of chemicals that are beneficial to human health and is consumed widely around the world [1]. However, because of the fast development of industry and agriculture in recent years, the quality of tea has begun to be affected by the concentration of heavy metals in tea plantations, tea processing methods, tea packaging methods, etc. Heavy metals such as lead (Pb), arsenic (As), cadmium (Cd), chromium (Cr), copper (Cu), and mercury (Hg) have considerable biological toxicity, which can be ingested by the human body when they are soaked or leached into the tea. The high concentration of various heavy metals in tea can threaten the health of tea drinkers. It can lead to cardiovascular, immune, and endocrine system diseases, and even cancer [2,3]. Chinese people consume about 1.2 × 10^9^ kg of tea each year; thus, heavy metals ingested through tea drinking may pose health risks. Therefore, understanding the status of heavy metal contamination in Chinese tea and the health risks associated with tea consumption are of great value to the maintenance of human health.

Studies on heavy metals in tea have been conducted in some famous tea-producing areas (such as Zhejiang, Fujian, and Guizhou, in China). Zhejiang is an important base for the production, export, and processing of green tea in China. Yan [4] assessed the heavy metal pollution in tea and tea garden soils in Songyang County, and the heavy metal concentrations in the tea and tea garden soils were within the safe range. In Fujian Province, tea is the most widely distributed cash crop [5]. As early as the 1990s, Fujian began to investigate the soils of tea plantations to provide a scientific basis for ensuring the sustainable development of regional agricultural production [5]. Guizhou province in southwestern China is also a major tea-growing province due to its suitable topography and is also a major tea-producing area for a high-quality tea named “Yunwu” [6]. Zhang, et al. [6] found that some areas in Guizhou have high concentrations of Cd and Hg, due to the generally high background values of soil heavy metals in Guizhou Province.

Soil and tea samples have been collected to determine heavy metal concentrations and evaluate pollution rates [7,8]. Some studies explored the relationship between heavy metals in soil and heavy metals in tea in a small tea garden [5,9,10], and evaluated the enrichment ability of different heavy metals by calculating enrichment factors (EFs) [8,11]. Additionally, the heavy metal concentrations and exposure risks of some famous teas such as Pu‘er tea and brick tea were also assessed [12,13,14,15,16,17]. These studies provided data on heavy metals in tea and soils of tea-producing areas throughout China for this study. However, studies on the nationwide distribution of heavy metal contamination in tea and the assessment of health risks associated with tea consumption are still lacking.

In this study, the heavy metal contamination status of tea in China was analyzed and the risk of heavy metal exposure from tea consumption was assessed using a meta-analysis method based on data collected from published papers. Firstly, the heavy metal concentrations in teas and tea plantation soils were collected from 227 published papers; the pollution status of different teas was analyzed by comparing with the national and World Health Organization (WHO) limits of heavy metal concentrations in tea; then, the spatial distribution of heavy metal concentrations in teas and tea plantation soils in China was obtained by spatial interpolation; and, finally, the risk of heavy metal exposure to tea consumers was assessed in terms of both non-carcinogenic and carcinogenic risk. Strengthening the detection of soil and tea pollution in tea plantations can better improve the quality of tea. The results of this study can provide a spatial distribution of heavy metal concentrations in tea and tea plantation soils across China, providing a reference for relevant authorities to develop a national land-use plan for tea cultivation and a scientific basis for ensuring sustainable development of regional agricultural production. In addition, understanding the overall situation of heavy metal concentrations in tea and the health risks can help the government to enact more appropriate food safety standards.

## 2. Materials and Methods

### 2.1. Data Collection of Heavy Metals in Tea and Tea Plantation Soils

Heavy metal concentrations in tea and tea plantation soils were collected from papers published from 1993 to 2021. These related papers were found by searching the CNKI/ISI website with the keywords tea or heavy metals of tea plantation soils, As/Cd/Cr/Cu/Hg/Pb, and China. There are also some papers from references of collected papers. Ultimately, 227 peer-reviewed articles (Table S1) related to tea and tea plantation soil heavy metals across China were collected. The concentrations of six kinds of heavy metals in seven types of tea (green, white, dark, black, oolong, scented, and yellow) and tea plantation soils were then collected. Considering that the consumption paths of tea in each region include both buying from the market and local cultivation, the data collected include both commercially available tea and tea cultivated in tea plantations.

If the regional administrative level of heavy metal concentration data collected is national, province, city, or county, then the data are considered “regional data”, and if it is township or village, the data are considered “point data” [18]. Table 1 shows the amount of data collected in the published papers on different heavy metals in tea and tea plantation soils and the national limits for different heavy metals.

### 2.2. Descriptive Statistical Analysis of Heavy Metals in Tea and Tea Plantation Soils

The overall status of heavy metal concentrations in tea and tea plantation soils and the pollution rates of heavy metals in different kinds of tea were obtained by statistical analysis on the collected data. When the heavy metal concentration of a sample was greater than the national standard, it was considered an above-standard sample. The pollution ratio was calculated using the number of samples that exceeded the standard:

(1) $$PR_{i}=\sum_{m=1}^{M}N_{i,m}/\sum_{m=1}^{T}N_{i,m},$$

where $PR_{i}$ is the pollution rate for tea *i. m* is the data record for this type of tea. *M* is the number of records of heavy metal concentrations above the standard. *T* is the total number of samples of heavy metal concentrations in tea *i*.

National limits in China for heavy metals in Table 1 are used to calculate pollution rates. Because most tea plantation soils are acidic [25], this study used the soil environmental quality standard of pH < 6.5 set out in the secondary standard of the “Soil Environmental Quality Standard” [20] as the reference to calculate the pollution rates in the tea plantation soil samples. In addition, the World Health Organization (WHO) limit values [21,23] for soil heavy metal concentrations were used to assess whether the quality of Chinese tea plantation soils meets international standards.

Tea can accumulate heavy metals in the soil where it is grown [26]. The roots of tea plants absorb the heavy metals from the soil. Then, the heavy metals are fixed and accumulate in the plant body. Therefore, the heavy metal pollution of tea planting soil will largely affect the heavy metal concentration of tea. An EF was used to assess the ability of tea in accumulating heavy metals from tea plantation soils [27]:

(2) $$\mathrm{EF}=(C_{t,i}/C_{ts,i})\times 100\%,$$

where $C_{t,i}$ is a recorded average concentration of heavy metal *i* detected in tea collected from different papers at one location, and $C_{ts,i}$ is its average concentration in the soil at the same location. The larger the value of EF, the higher the possibility that heavy metal *i* in the tea comes from the soil of the tea plantation.

### 2.3. Spatial Interpolation of Heavy Metals in Tea and Tea Plantation Soils

Kriging interpolation is a geostatistical method that uses spatial autocorrelation to build a spatial model around sampling points. Kriging methods have been frequently used to obtain the spatial difference in heavy metal concentrations at a regional scale [28,29,30]. We followed the method proposed by Zhang et al. (2021) [18] to attain the distribution of heavy metals in tea and tea plantation soils across China.

The collected heavy metal concentration data were first assigned to descending administrative levels. If the data collected were at the national level, they were assigned to the 34 provincial centroids across China, and so on, with the lowest administrative level being the township level, whereby, eventually, the collected heavy metal concentration data can be presented in point form across China. Based on these point data, the traditional Kriging interpolation method was used to obtain the spatial distribution of heavy metal concentrations in Chinese tea and tea plantation soils. The MAE and the RMSE were used to evaluate the interpolation results.

### 2.4. Health Risk Assessment

A model to analyze human exposure to heavy metals during tea consumption was developed by the United States Environmental Protection Agency (EPA) and the Dutch National Institute of Public Health and Environmental Protection. This method evaluates human health exposure risk through three main pathways: dermal exposure, respiratory system, and oral ingestion. The main exposure route of tea drinking is oral intake [17], so this study only analyzed the exposure risk of oral ingestion. The adults were considered to be the exposed population in this study.

The estimated heavy metal daily intake (EDI) is the basic variable of the health risk assessment. EDI is calculated as follows [31]:

(3) $$\mathrm{EDI}=\left(C\times E_{F}\times E_{D}\times F_{I}\times F_{R}\right)/(W_{AB}\times T_{A}\times 1000),$$

where C is the average content of a certain heavy metals in tea (mg/kg).$\;E_{F}$ refers to exposure frequency ($E_{F}$ = 365 days/year). $E_{D}$ is exposure duration ($E_{D}=70$ years).$\;F_{I}\;$ is the ingestion rate of tea (g/(person·day)). $F_{R}$ is a processing factor in the exposure system, referring to a leaching rate of heavy metals during the tea infusion [32,33]. $W_{AB}$ is standard body weight (the average weight of Chinese adult men (66.2 kg) and women (57.3 kg), $W_{AB}=61.7$ kg), and $\;T_{A}$ is the average exposure time ($T_{A}$ = $E_{D}\;\times$ 365 days for non-carcinogens; $T_{A}$ = 70 × 365 days for carcinogens) [34].

According to statistics, the average publication year of collected papers was 2015, and the average time interval between the publication of the papers and data collection was 3 years. Therefore, the data collected in this study reflect the situation in 2012 at an average level. Subsequently, based on the proportion of tea consumption in various regions calculated from the 2009 Tea Yearbook [35] and the total consumption of tea recorded in the Tea Statistical Yearbook in 2012 [36], the consumption of each province in 2012 was obtained. Because the Statistical Yearbook lacks statistics for Taiwan Province, Hong Kong, and Macau, the average $F_{I}$ of all other provinces was used.

The non-carcinogenic risk for a specific metal in tea was evaluated by the hazard quotient (HQ) calculated by Equation (4) [31]:

(4) HQ=EDI/RfD,

where RfD is the EPA-recommended daily heavy metal intake limited dose (mg/(kg·d)) or a provisional tolerable weekly intake defined by the World Health Organization (Table S2) [37].

This study used a hazard index (HI) to assess the overall risk potential of non-carcinogenic effects [31]. It is the sum of HQ of all heavy metals studied.

(5) $$\mathrm{HI}=\sum_{i=1}^{n}HQ_{i},$$

where $HQ_{i}$ is the *HQ* of heavy metal *i*. If the HI exceeds 1, there is a probability of observing non-carcinogenic health effects, and this probability increases as the HI value increases [38].

Since not every heavy metal is carcinogenic, according to the classification of the International Agency for Research on Cancer (IARC) [39], only the carcinogenic risks of As, Cd, Cr, and Pb, which have cancer slope factor ($SF_{i}$), were calculated in this study. The carcinogenic risk for a specific metal in tea was evaluated by Equation (6), and the total risk is the sum of the risks of all heavy metals calculated using Equation (7) [38]:

(6) $$Risk_{i}=EDI_{i}\times SF_{i},$$

(7) $$Risk_{total}=\overset{n}{\underset{i=1}{\sum }}Risk_{i},$$

where $Risk_{i}$ is the health risk of a specific carcinogenic metal *i*. $SF_{i}$ is the cancer slope factor of a specific carcinogenic metal *i* (Table S2) [38]. The acceptable carcinogenic risk level is 10^−6^–10^−4^ [40].

## 3. Results and Discussion

### 3.1. Overall Status of Heavy Metals in Tea and Tea Plantation Soils

**Figure 1:**
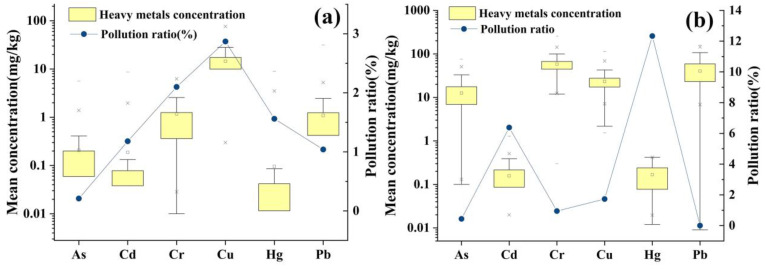
(**a**) Boxplot figure of heavy metal concentrations in teas and pollution rates of heavy metals in (**a**) teas; (**b**) soils. (The square box inside the box represents the mean value of the data. The ‘x’ marks outside the whisker range represent the data points that fall within the 1st percentile (1%) and the 99th percentile (99%) of the data distribution. The hyphen marks indicate the extreme values).

Statistical analysis on heavy metal concentrations in tea and soils was carried out, and the contamination rates were calculated based on the limit values of various heavy metals in Table 1 (shown in Table S3 and Figure 1). The detection rate of all the heavy metals except Cr in the collected tea samples was not 100% (the lowest concentration was zero) (Figure 1a). The maximum values of Cr and Pb concentrations in tea were more than eight and six times the standard values, respectively. The reason for such high Cr and Pb concentrations in some studies might be due to the addition of lead chrome green by the merchant to make the tea look fresher and for tea processing [41]. The different pollution rates of heavy metals in tea were not very serious with all the contamination rates below 3%. The contamination ratio of heavy metals in tea is ranked as Cu > Cr > Hg > Cd > Pb > As; therefore, routine inspection and frequent measurement of Cu and Cr in commercially available tea is required.

The detection rate of all heavy metals except Cd in the collected soil samples from tea plantations was 100% (lowest value > 0) (Figure 1b). The maximum Cd in tea plantation soils was more than four times that of standard values, because Cd is stable and easy to be accumulated in soil [42]. The pollution ratio of Hg in the tea plantation soil samples is more serious than that of other heavy metals and may be caused by the application of too many pesticides containing Hg during planting management [43]. The Pb concentration did not exceed the standard in any tea plantation soil samples; however, the contamination rate of Pb in tea was 1.04%. This indicates that there are other sources of Pb contamination in tea besides the planted soil. Previous studies [44] have shown that the Pb in tea mainly comes from atmospheric deposition, and the Pb contribution rate of automobile exhaust emissions in young tea leaves is nearly 80%, and that in old tea leaves is nearly 60%. The pollution rates 2 (as shown in Table S3) reflect the international level of soil quality in Chinese tea plantations. Since the WHO limits for soil heavy metal concentrations are much lower than those in China (Table 1), almost all the data collected on soil heavy metal concentrations in tea plantations were at heavy contamination levels. According to previous studies [45], irrigation of crops with untreated wastewater can lead to the accumulation of heavy metals in the soil, so as industrialization continues, the treatment of industrial wastewater should also be tightly controlled.

### 3.2. Pollution Rates of Different Heavy Metals in Different Types of Tea

**Figure 2:**
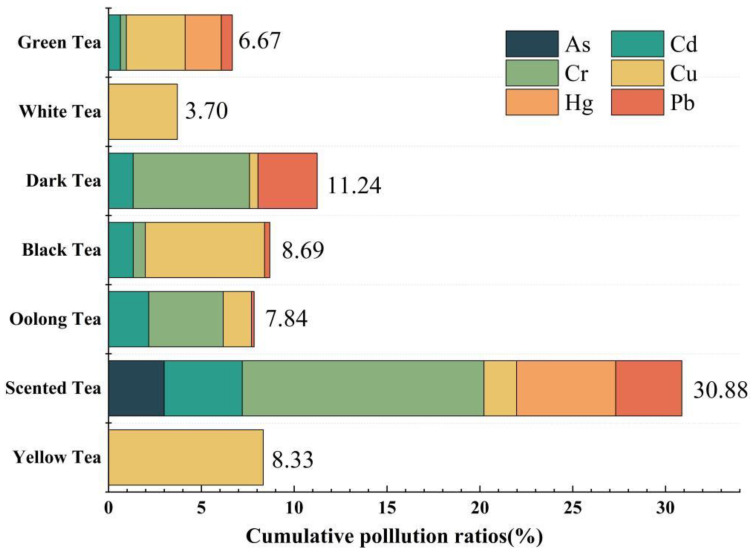
Cumulative pollution rates of heavy metals in seven types of tea.

The teas were classified into seven categories based on differences in manufacturing methods and quality. The average concentrations of heavy metals and their pollution rates in different types of tea are described in Table S4. Figure 2 shows the cumulative pollution rates of heavy metals in seven kinds of tea. Green tea is the earliest produced and the most famous type of tea in China. The quality of green tea samples collected was good, and the heavy metal content rarely exceeded the standard. However, heavy metal pollution was very severe in the scented tea, although the sample size of the scented tea was small, which might have influenced the average heavy metal concentrations. The concentration of each heavy metal in some scented tea samples exceeded the standard. This is mainly because scented tea is often sprayed with chemical pesticides, which contain heavy metals, during the growth process [41,46]. The total pollution rate of the other teas was between 5% and 10%. The heavy metal pollution rates of white and yellow teas were the lowest, with only Cu exceeding the limits.

The Cu contamination in teas may be related to the extensive use of organic fertilizers in tea plantation soils. Cu is an essential trace element that plays an important role in human metabolism but should be consumed in moderation. Cd had the second-highest frequency of contamination among the seven studied teas. Cd compounds can be absorbed by the body through breathing and accumulate in the liver or kidneys, causing damage. It also causes osteoporosis and bone softening [47], and its contamination rate is higher in scented and oolong teas. Therefore, these two teas should be consumed in moderation by the elderly and children. Pb was contaminated in five kinds of teas, and its contamination rate was higher in black tea and scented tea. Cr is involved in human sugar and fat metabolism and has key physiological functions for human health [48], but some cancers may be associated with chronic chromium exposure. Black and scented teas have heavy concentrations of Cr; therefore, people should be careful not to swallow the tea leaves when drinking these teas. Hg and As can harm the human skin and respiration, as well as the digestive, urinary, cardiovascular, neurological, and hematopoietic systems. They are toxic substances that readily cross the blood–brain barrier [49] and can be hazardous to human health when ingested in excess over time. Fortunately, these two heavy metals were not excessive in most of the collected tea samples.

### 3.3. Spatial Distribution of Heavy Metals in Tea across China

**Figure 3:**
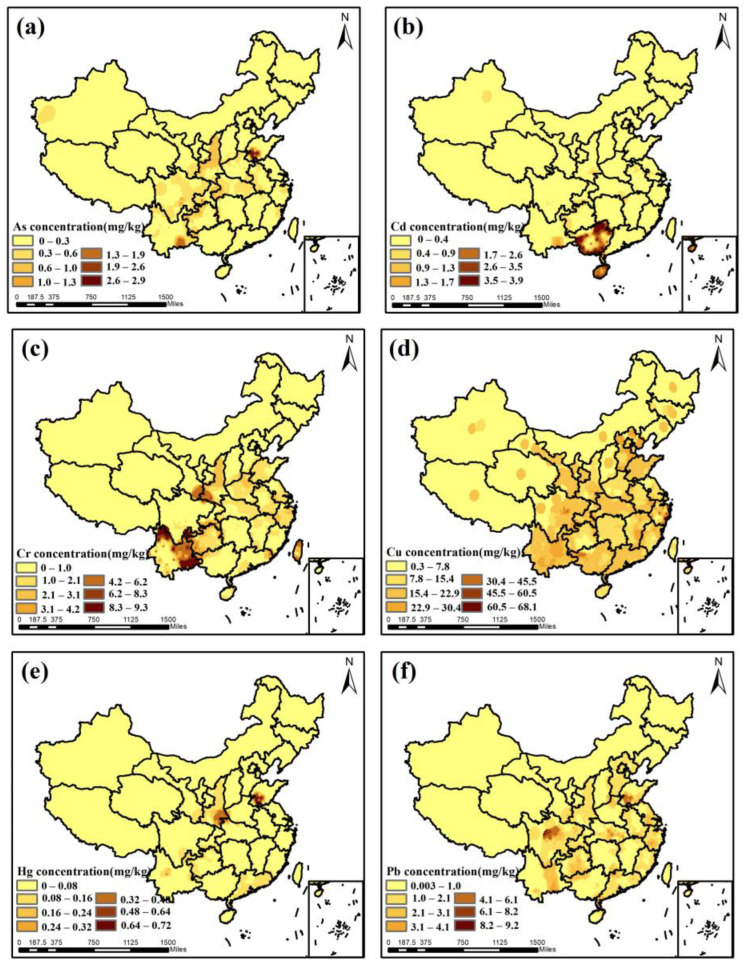
Spatial distribution of heavy metal concentrations in tea: (**a**): As; (**b**): Cd; (**c**): Cr; (**d**): Cu; (**e**): Hg; (**f**): Pb.

An ordinary Kriging method was performed to acquire the spatial distribution of heavy metals in tea and tea plantation soils (Figure 3 and Figure S1). To investigate the causes of the accumulation of heavy metal concentrations in tea, this study screened sample data of heavy metal concentrations in tea and tea garden soil located at the same location to calculate the EF of different heavy metals, and the results are shown in Figure S2. As shown in Figure S2, the EF averages of six heavy metals in tea are sorted below: Cu > Cd > Hg > Pb > As > Cr. This suggests that Cu, Cd, and Hg have a higher enrichment capacity.

Comparing Figure 3 with Figure S1, the spatial distribution of high Cd and Hg concentrations in tea is not consistent with the distribution of high Cd and Hg concentrations in tea garden soils, except that Cu concentrations in tea and tea garden soils are at high levels nationwide. This suggests that the heavy metal concentrations absorbed by tea leaves from tea plantation soil do not positively correlate with the total amount of heavy metals in the tea plantation soil. The amount of heavy metals in the soil that can be used by tea leaves (bioavailability) [26] is influenced by many other physicochemical properties. The areas with high As values were mainly located in southwestern Shandong Province and southeastern Yunnan Province (Figure 3a). Based on studies [50,51], the high As concentrations in Shandong and Yunnan regions were due to the high As concentrations in local scented teas, which are also noted to have the highest rates of heavy metal contamination in Section 3.2. Areas with serious Cd pollution are mainly in southwest China (Figure 3b), due to the strong Cd uptake and enrichment capacity of “Kuding tea”, a specialty tea grown in Hainan and Guangxi Provinces [52]. The areas with severe Cr pollution are concentrated primarily in southwestern China and the southern parts of Shaanxi and Gansu Provinces (Figure 3c). The high values in these regions are also caused by scented teas, and the article [53] also mentions that the dissolution rates of various elements in teas are related to the binding state of trace elements. The concentration of Cu in tea is high nationwide (Figure 3d). This may be because metal machinery and equipment containing copper are often used in the production and processing of tea in various locations. Areas with serious Hg pollution were mainly in southeastern China and Shaanxi Province (Figure 3e). This is also caused by scented teas [51,54]. The areas with serious Pb pollution are mainly in the eastern coastal areas of China and Sichuan Province (Figure 3f). The study [55] suggests that the concentration is higher in the large green tea-consuming provinces on the eastern seaboard because of the artificial addition of lead and chromium green in the sale of green tea by some traders. The higher concentration of Pb in tea in Sichuan Province is due to the high concentration of heavy metals in the soil of the region [56].

Overall, the concentrations of As, Cd, and Pb in tea in most areas of China do not exceed half of the national limit values. Cr and Hg are highly polluted in localized areas, while Cu pollution is regional. The regions with frequent high values of heavy metal concentrations are the eastern and southwestern parts of China and Shaanxi Province. It is clear from the analysis that the concentration of heavy metals in tea is not positively correlated with the total amount of heavy metal in the soil of the tea plantation. Although the total amount of heavy metals in the tea garden soil is the main focus of our research, other factors also need to be considered, including the variety of tea, the physicochemical properties of the soil, and the plant availability of heavy metals in the soil [57].

### 3.4. Health Risk Assessment of Tea Consumption across China

**Figure 4:**
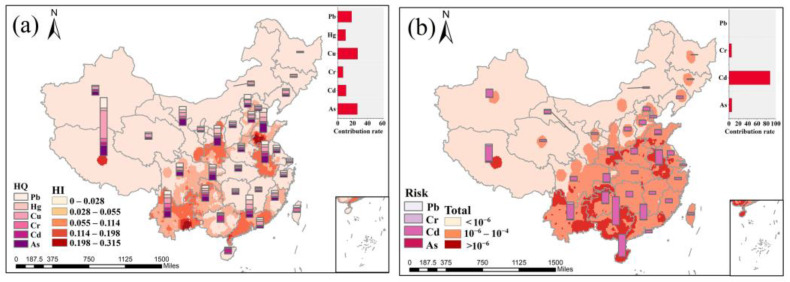
Spatial distribution of (**a**) hazard index of tea heavy metals for adults and (**b**) carcinogenic risk of tea heavy metals (As, Cd, Cr, and Pb) for adults. (The histogram of the stack shows the contribution of different heavy metals to the health risk).

Non-carcinogenic and carcinogenic risks were evaluated separately for male adults and female adults in this study, based on the mean weight of the different genders. Tables S5–S8 show the results of the health risk assessment for different provinces, and the heavy metal concentrations in tea used in the calculation are the average values for each province. The distribution of the hazard index of tea heavy metals and the carcinogenic risk of tea heavy metals (As, Cd, Cr, and Pb) for adults (Figure 4a,b) were obtained by using the spatial distribution of heavy metals in tea in Section 3.3.

The national average HI was about 0.04, and all of the HIs in the 34 provinces and regions were below 1, indicating that they are low or negligible risk and do not pose a significant non-carcinogenic risk (Tables S5 and S6). The risk for women was slightly high due to their weight, but still at a low level. As can be seen in Figure 4a, the distribution of non-carcinogenic risks for heavy metals is generally consistent with the distribution of heavy metal concentrations in tea, with high values occurring in Shandong Province, Shaanxi Province, and the southwestern part of China. It is worth noting that Tibet has the highest HI. Although the tea concentrations in this region are not high, the consumption of tea by the Tibetan people is great. For the residents of the Tibetan plateau, tea is an indispensable part of daily life. For Tibetans who live in high-altitude cold areas for a long time and eat beef and mutton, tea can relieve constipation, promote urination, and lower blood lipids. However, the average HI of Tibet was only 0.18, which was much lower than 1, suggesting that the daily drinking of tea poses few health risks to humans in Tibet. The HQ of the different heavy metals ranked: Cu > As > Pb > Cd > Hg > Cr. Therefore, Cu, As, and Pb are the main non-carcinogenic risk factors.

Tables S7 and S8 show that the carcinogenic risks for As, Cr, and Pb are all within acceptable limits, but the carcinogenic risks for Cd are unacceptable in many provinces. As with non-carcinogenic risks, the risk of cancer is somewhat higher for women than for men due to differences in weight. The higher carcinogenic risks are mainly found in Anhui Province, southern Shaanxi Province, and southwestern China, broadly in line with the non-carcinogenic risks. Cd is the main carcinogenic factor that needs to be controlled (Figure 4b).

In general, the carcinogenic risk of metals is more serious than the non-carcinogenic risk, and the exposure risk is higher for women than men. Cd, As, Cu, and Pb should receive more attention and control from the government. Some areas where the carcinogenic risk of Cd is beyond the acceptable range should find the source of contamination as soon as possible and adjust agricultural cultivation strategies and land-use planning to safeguard the dietary health of residents.

## 4. Uncertainties and Implications

Tea heavy metal concentrations, total tea intake, and different heavy metal leaching rates during tea infusions were considered when evaluating the health risk of Chinese tea drinking; thus, any uncertainties in these three factors also posed uncertainties in the evaluation results.

The first uncertainty is the tea heavy metal concentrations. Because heavy metal concentrations in tea were attained from disparate papers, the differences in sample processing approaches and concentration measurement approaches may affect the consistency of heavy metal concentrations, although only the concentrations that were supported by strict quality control procedures were adopted in this research. The research collected heavy metal concentrations in papers from two major databases from 1993 to 2021, covering the whole of China. Through our calculations, the average year of publication of the articles was 2015. In addition, the time interval between the time when the concentration data were measured and the time when the paper was finally published was calculated to be 2.8 years. Therefore, this study presents the spatial distribution of heavy metal concentrations in tea in approximately 2012. In fact, unless there is a large exogenous contamination, there is no significant inter-annual variation in heavy metal concentrations in agricultural products [18]. Therefore, this study is reliable in assessing the current status of heavy metal contamination and exposure risk by using comprehensive heavy metal concentration data from tea and tea plantation soils for more than 20 years.

The second uncertainty is the ingestion rates of tea. Because the Tea Statistics Yearbook only contains tea consumption for the whole of China in 2012 [36], this study used the tea consumption of China’s provinces in 2009 [35] to calculate the proportion of tea consumption in each province in the country and then calculated the tea consumption of China in 2012. Because the tea-drinking habits of various provinces in China are unlikely to change greatly within 3 years, it is believed that any errors in this calculation method are small. However, because the statistical yearbook lacks data for Taiwan Province, Hong Kong, and Macau, the data for these three regions were replaced by the average value of other Chinese provinces in this study. Therefore, the health risk assessment results of these three regions may contain large errors.

The third uncertainty is the leaching ratios of heavy metals in different tea liquids. The leaching ratios of heavy metals in tea are affected by factors such as the type of tea, the time the tea brews, the temperature of the brewing tea, and the amount of brewing tea. In this study, the average value of the heavy metal leaching rates of different types of tea steeped for 30 min was used because this is consistent with the tea-drinking habits of most people. Therefore, the results of this study have certain limitations. With high-quality data on the leaching rates of heavy metals at different soaking times using different types of tea, the study results could be further optimized to provide more accurate health risk assessments for all provinces in China.

## 5. Conclusions

In this study, the health risks of being exposed to heavy metal concentrations in teas across China were evaluated. The heavy metal concentrations in tea and soil concentrations were collected. The results showed that most of the heavy metal concentrations in teas were under the limits enacted by the Chinese government. Among the seven types of tea studied, scented tea had the most serious heavy metal contamination.

The heavy metal concentrations in teas do not only depend on the total concentration of heavy metals in the soil concentration, but also tea varieties, soil physicochemical properties, and heavy metal binding states will affect the bioavailability of heavy metals in soil.

The health risks of tea consumption were evaluated for residents in the 34 provinces in China. The Tibetan region had a high level of both non-carcinogenic and carcinogenic risk in the country due to its higher consumption. Overall, the carcinogenic risk of metals was more severe than the non-carcinogenic risk, with women having a higher exposure risk than men. As, Cu, and Pb were the main non-carcinogenic risk factors and Cd was the main carcinogenic risk factor. The carcinogenic risks of Cd in Shaanxi Province, Anhui Province, and southwest China are beyond the acceptable safe range, and heavy metal pollution in these areas should be strictly controlled.

## Figures and Tables

**Table 1:** Number of collected samples and the limit values of heavy metal concentrations in tea and tea plantation soils.

Elements	Tea Samples		Tea Plantation Soil Samples	
	Number of Samples	Limit Value (mg/kg)	Number of Samples	Limit Value (mg/kg) (China/WHO)
As	4803	2 [19]	6767	40 [20]/NG
Cd	5257	1 [19]	7435	0.3 [20]/0.003 [21]
Cr	4903	5 [19]	6722	150 [20]/0.1 [21]
Cu	4881	30 [22]	6772	50 [20]/NG
Hg	4026	0.3 [19]	6424	0.3 [20]/0.05 [23]
Pb	6611	5 [24]	7181	250 [20]/0.1 [21]

## Data Availability

Data set is available on request to corresponding authors.

## Supplementary Materials

The following supporting information can be downloaded at: https://www.mdpi.com/article/10.3390/toxics11080662/s1, Table S1: Published papers on Heavy metal concentrations in tea collected in this study; Table S2: Average daily exposure reference dose and cancer slope factor of various heavy metals; Table S3: Concentration of heavy metals and pollution rates in tea and tea plantation soils; Table S4: Heavy metal concentrations and pollution ratios in different kinds of tea; Table S5: The non-carcinogenic risk assessment of tea consumption for male adults in China; Table S6: The non-carcinogenic risk assessment of tea consumption for female adults in China; Table S7: The carcinogenic risk assessment of tea consumption for male adults in China; Table S8: The carcinogenic risk assessment of tea consumption for female adults in China. Figure S1: Spatial distribution of heavy metal concentrations in tea plantation soils; Figure S2: The number of samples from the same location used for different heavy metals and the calculated EF averages.
