# Supplementary material for: Current Status and Health Risk Assessment of Heavy Metals Contamination in Tea across China
Source: Toxics. 2023 Aug 1;11(8):662. doi: 10.3390/toxics11080662 (PMC10459165; doi:10.3390/toxics11080662)
Supplement: Supplementary file 1 [file toxics-11-00662-s001.zip › toxics-2481498-supplementary.pdf]

Current Status and Health Risk Assessment of Heavy Metals Contamination in Tea across China

Chenglin Hu[1,2,3] , Xiuying Zhang[1, *], Nan Zhan[1], Youcun Liu[4, *]

[1] International Institute for Earth System Science, Nanjing University, Nanjing 210023, China

[2] Key Laboratory of Urban Land Resources Monitoring and Simulation, Ministry of Natural Resources, Shenzhen 518000, China

[3] Jiangsu Center for Collaborative Innovation in Geographical Information Resource Development and Application, Nanjing 210023, China

[4] School of Geography and Tourism, Jiaying University, Meizhou 514015, China

*Correspondence: zhangxy@nju.edu.cn (X.Z.); liuyoucun@hotmail.com (Y.L.)

Table S1 Published papers on Heavy metals concentrations in tea collected in this study

Num-ber	Reference	Journal	Publish year
1.	Investigation and analysis of heavy metals and rare earth elements content in 2 kinds of Chinese holly leaf	Journal of Food Safety and Quality	2017
2.	Drinking safety evaluation of 3 kinds of mulberry leaf dark tea	Science of Sericulture	2019
3.	Investigation of heavy metals content and pesticide residues in 4 kinds of herb teas	Journal of Food Safety and Quality	2016
4.	Measurement and analysis of harmful elements and pesticide residues in seven different types of chrysanthemum	Cereal and Food Industry	2020
5.	Investigation and analysis of heavy metal pollution in tea in Jiangxi province from 2014 to 2016	Experimental and Laboratory Medicine	2020
6.	AAS determined the heavy metal content in Wuzhi Shan Kuding tea	Food Engineering	2012
7.	Health risk assessment of heavy metals in soil-tea system from Tieguanyin Tea Garden of Anxi County	Food Industry	2021
8.	Analysis of heavy metal content in Paeoniae Radix Alba at Bozhou	Journal of West Anhui University	2014
9.	Analysis of heavy metal content in Shangluo tea leaves in different origins and harvest seasons	Shaanxi Journal of Agricultural Sciences	2015
10.	Comparative study on the contents of heavy metals in marigold flowers and soil from different producing areas	China Food Additives	2020
11.	Changes in tea biochemical indexes and soil physicochemical properties at different altitude ranges of Lushan Mountain	Journal of Anhui Agricultural University	2017
12.	Analysis of the heavy metal contents in different teas and tea water	Journal of Jilin Medical College	2015
13.	Tea quality and safety risk assessment analysis—Take Tongren City as an example	Cultivation and breeding	2018
14.	Analysis and hazard evaluation of aluminium, lead, arsenic, mercury, chromium and cadmium content in tea	Journal of Food Safety and Quality	2021
15.	A study on the contents of Iron, Zinc, Copper in common Tea leaves	Journal of Quanzhou Normal University	2017
16.	Determination and analysis of heavy metal elements lead, chromium, cadmium, and arsenic mercury in tea leaves in Chizhou City	South China Agriculture	2019
17.	Survey on the content of metal elements in the black Tea produced in Duyun	Guangzhou Chemical Industry	2015
18.	Pollution evaluation of heavy metals in soil of tea plantations and tea at Emeishan and Jiajiang	Journal of Anhui Agriculture	2016
19.	Determination of 10 Kinds of harmful elements in Fanjing mountain Green Tea	Food Research And Development	2017
20.	Determination of heavy metal content and research on enrichment of tea cultivars in Fujian Area	Modern agricultural technology	2019
21.	Study on heavy metal content in four kinds of tea in Fujian Province	Anhui Agriculture Science Bulletin	2018
22.	Eco−geochemical characteristics of the Tieguanyin tea gardens in Fujian Province	Geology in China	2015
23.	Analysis of 17 kinds of inorganic elements and 5 kinds of heavy	Food Safety Guide	2018

	metal safety evaluation in Guzhang Maojian and 3 kinds of alternative tea		
24.	Study on the remediation effect and environmental benefits of ornamental plants on heavy metal soil:A case study of Kaifeng City chrysanthemum	Environmental Chemistry	2020
25.	Studies on Composition and Acute Toxicity Test of Loquat Flower Tea	Southeast Horticulture	2016
26.	Research of Pollution and Enrichment of Heavy Metal in Soil and Tea in Typical Tea Producing Areas of Guizhou Province	Environmental Science&Technology	2012
27.	Heavy metal accumulation and leaching characteristics of Niaowang tea in Guizhou	Guangdong Agricultural Sciences	2013
28.	Analysis of Trace Elements in Niaowang Tea from Guizhou Province	Guizhou Science	2012
29.	Monitoring and Pollution Assessment of Heavy Metals in Soil and Tea in Shiqiantai Tea Producing Area of Guizhou Province	Journal of Agricultural Catastropholgy	2020
30.	Analysis of trace elements and heavy metal components in different parts of two kinds of golden flower tea native to Guizhou	Agriculture and Technology	2020
31.	Distribution of Heavy Metals in Soil and Tea from Yunwu Tea Area in Guizhou Province and Diffusion Characteristics of Heavy Metals in Tea Infusion	Food Science	2013
32.	Evaluation of heavy metals and rare-earth elements in famous black teas at home and abroad	Journal of Food Science and Technology	2017
33.	Investigation of heavy metal content of different types of tea in Hunan Province and its pollution evaluation	Zhejiang Agricultural Sciences	2020
34.	Analysis of the current situation of pesticide residues and heavy metal pollution in tea in Hunan Province	China Food Safety	2021
35.	The cadmium pollution investigation of tea trees and soil in northeastern Hunan province tea gardens	Chinese Journal of Health Inspection	2015
36.	Study on lead and cadmium pollution of tea heavy metals in northeast Hunan Province	Master's degree thesis of Hunan Agricultural University	2015
37.	Study on the contents of heavy metals in whole Huai Chrysanthemum	Applied Chemical Industry	2016
38.	Health risk assessment and extraction behavior of heavy metals in local tea of Huangshan	Food Industry Technology	2017
39.	Comparative analysis of the contents of three heavy metal elements Cr, Ni, Co in Jiangxi Famous Teas	Acta Agriculturae Universitatis Jiangxiensis	2020
40.	Pollution evaluation of heavy metals in tea and soil of tea plantations in Jinghong City	Journal of Anhui Agriculture Science	2011
41.	Analysis on mineral elements of camellia sinensis plantations in the Qingshuijiang of Leishan County	Journal of Sichuan Agricultural University	2017
42.	Health risk assessment of fluorine and heavy metals in LiuPaoTea	Food & Fermentation Technology	2017
43.	Concentrations and health risk assessment of heavy metals in tea garden soil and tea-leaf from a mine county in central Fujian	China Environmental Science	2018

	province		
44.	A case study of cadmium distribution in soil-tea plant-tea soup system in central Fujian province and relative health risk assessment	Journal of Tea Science	2018
45.	Analysis of the content of lead and cadmium in jasmine tea	Fujian tea	2016
46.	Determination of seven trace elements in Loquat scented Tea	Technology & Development of Chemical Industry	2017
47.	Determination and analysis of chemical compositions on dandelion tea and leaves	Science and Technology of Food Industry	2014
48.	Determination and risk assessment of heavy metal elements Arsenic, Lead, Cadmium, Chromium, Mercury And Copper in Pu'er Tea	Modern Food	2020
49.	Concentrations and diffusion characteristics of heavy metals in green tea samples from urban markets in Guizhou Province	Modern Food Science and Technology	2020
50.	Regional differences of heavy metals in soil and tea in tea areas of South Guizhou	Guizhou Agriculture Science	2012
51.	The main tea producing areas in Qianxi prefecture and evaluation of Lead, Copper, Arsenic content determination	Modern Food	2017
52.	Study on the morphological distribution and bioavailability of heavy metals in soil of green tea gardens in Rizhao City	Master's thesis of Qufu Normal University	2018
53.	Analysis of tea quality and assessment of heavy metal content status in tea plantations of southern Shaanxi Province, China	Transactions of the Chinese Society of Agricultural Engineering	2020
54.	Analysis and pollution evaluation of soil heavy metal content in tea gardens in Xixiang County, Shaanxi Province	Shaanxi Journal of Agriculture Sciences	2017
55.	Study on content and distribution characteristics of heavy metals elements in tea and soil of Shangluo	Tianjin Agricultural Sciences	2013
56.	Enrichment characteristics of metal elements in leaves from four species of yellow flower camellia	Guihaia	2016
57.	Heavy metal content of Tieguanyin tea leaves and their health risk assessment	Journal of Jilin University(Earth Science Edition)	2015
58.	Wuzhou Liupao tea soil raw materials tea and finished tea heavy metal content monitoring and safety evaluation	Agriculture and Technology	2016
59.	Contents and enrichment coefficents of heavy metals in sediment and lotus grown in ponds at downtown Wuyishan city	Fujian Journal of Agricultural Sciences	2018
60.	Factors Affecting Bioavailability of Chromium, Zinc and Nickelin Soil at Wuyi Rock Tea Plantations	Fujian Journal of Agricultural Sciences	2019
61.	Evaluation of the quality of green tea products and their pollution status in southwest China	Southwest China Journal of Agricultural Sciences	2000
62.	Content of rare earth and heavy metal elements in xinyang maojian tea and their risk assessment	Guizhou Agricultural Sciences	2017
63.	Investigation of tea's heavy metal pollution and correlation with soil in Ya'an	Sichuan Agricultural University Master's degree Dissertation	2014
64.	Investigation and evaluation of heavy metals in tea of FengHuang Moutain tea gardens in east Guangdong Province	Environmental Science＆Technology	2010
65.	Heavy metal pollution of agricultural products:present situation in	Chinese Agricultural Science Bulletin	2017

	some areas of Yunnan		
66.	Investigation on heavy metal content in large leaf species of Pu’er Tea in Dali, Yunnan Province	Journal of Kunming University	2017
67.	Health risk assessment of Cu，Pb，Zn，Cd，Cr and As in tea leaves from Fengqing County in Yunnan	Journal of Kunming University	2019
68.	Distribution characteristics of heavy metal content in tea leaves in different seasons from Fengqing tea plantation in Yunnan	Journal of Kunming University	2021
69.	Health risk assessment of health metals in Pu’er Tea of Yunnan Menghai	Journal of Kunming University	2020
70.	Analysis on leaching characteristics of heavy metals in tea from Yunnan Baoshan	Journal of Kunming University	2016
71.	Product quality analysis of Jing-mai ancient mountain Pu'er tea in Yunnan province	Food Research And Development	2018
72.	Analysis on quantity of heavy metals and total rare earth elements in tea leaves from Yunnan Pu’er	Journal of Kunming University	2015
73.	Exposure levels of lead, selenium, arsenic and mercury in brick-teas from main producing areas in China	Journal of Hygiene Research	2017
74.	The monitoring and pollution evaluation of heavy metals in the soil and tea in tea gardens in Songyang County, Zhejiang Province, meets the national safety requirements	Beijing Agriculture	2014
75.	The monitoring and evaluation of heavy metal contents in the tea garden soil and the tea in the typical tea garden of Zhenjiang	Hubei Agricultural Sciences	2014
76.	Detection of biochemical components and quality analysis of ancient tea tree black tea of zhuquesi in Ziyang population species	Hubei Agricultural Sciences	2020
77.	Study on nutritional components of three kinds of Fanjing mountains tea	Food Research And Development	2017
78.	Determination of 14 elements in five kinds of ripe Pu’er Tea and health risk assessment of heavy metals	Journal of Yuxi Normal University	2020
79.	Analysis of trace elements of 5 kinds of Anhui green tea	Journal of Anhui Tcm College	1998
80.	Investigation and analysis of heavy metal pollution in tea in Jiangxi Province from 2014 to 2016	Experimental and Laboratory Medicine	2020
81.	Analysis and risk assessment of lead, cadmium, arsenic and mercury contamination in teas sold in Shandong Province in 2018	Chinese Journal of Health Inspection	2021
82.	Variation patterns of five metal elements contents in crush, tear and curl black tea during manufacturing process and exploration of visual rapid detection method of Chromium	Chinese Academy of Agricultural Sciences Dissertation	2018
83.	Determination of eight trace elements content in Pekoe tea from Lingyun County, China by flame atomic absorption spectrometric	Guangdong Chemical Engineering	2015
84.	Determination of the dissolved rates of eight trace elements in seven kinds of scented teas	Chinese Journal of Spectroscopy Laboratory	2010
85.	ICP - AES for the determination of trace elements in Qingdao Laoshan green tea	Chinese Journal of Spectroscopy Laboratory	2011
86.	Detection method of heavy metals in tea soup by ICP - AES	Environmental Science and Technology	2009

87.	Determination of mineral elements in two grades of three green Tea varieties by ICP - AES	Spectroscopy and Spectral Analysis	2011
88.	Determination of nickel in green tea by ICP − AES	Guangzhou Chemical Industry	2020
89.	Determination of ten trace elements in eight herbal flowers and their infusions from Yunnan province, China by ICP - AES	Journal of Henan Institute of Science and Technology	2014
90.	ICP - MS determination of 16 elements in Suzhou Taihu Dongting Mountain black tea and tea soup	Quality and safety of agricultural products	2019
91.	ICP - MS study of 6 methods of quantitative analysis of heavy metals in tea	Analytical instruments	2012
92.	Investigation of elemental concentration and extraction rate of Green Tea by ICP - MS	Hebei Journal of Industrial Science and Technology	2015
93.	Determination of heavy metals in 30 Batches of Carthami Flos by ICP - MS	Guangzhou Chemical Industry	2016
94.	Determination of heavy metals and Harmful Elements in Xanthii Fructus by ICP - MS	China Pharmacist	2019
95.	Determination of 13 kinds of elements in Roselle by ICP - MS WEI Jiangchen	Modern agricultural technology	2019
96.	Determination of Lead, Mercury, Cadmium, Arsenic, Copper heavy metals in weight-reducing tea by ICP - MS	Northern Pharmacy	2013
97.	Determination of dissolving-out characteristics of elements in Ganpu tea by inductively coupled plasma mass spectrometry	Chemical Analysis and Meterage	2019
98.	Determination of ten metal elements in drinking water, green tea and rice by ICP - MS	Food Research And Development	2013
99.	Analysis of leaching rates of 8 heavy metals in Mogan Yellow Bud Tea by Inductively Coupled Plasma Mass spectrometry	Modern Food	2021
100.	Analysis of 9 heavy metal elements in Xinyang Maojian Tea Using Inductively Coupled Plasma Mass spectrometry	Food Industry	2017
101.	Simultaneous determination of 18 elements in tea by ICP - MS	Modern Preventive Medicine	2016
102.	ICP - MS Detection of Lead in Food, Arsenic, Mercury, Cadmium, Barium, Chromium, Silver, Nickel 8 Harmful Elements	Food Industry	2015
103.	Determination of trace elements and harmful heavy metals in tea by ICP − OES	Journal of Anhui Agricultural University	2021
104.	Determination of seven heavy metal elements released from green tea by ICP – OES	Chemical Analysis and Meterage	2014
105.	Determination of 26 mineral elements in golden tea samples by using ICP − OES and ICP − MS	Studies of Trace Elements and Health	2013
106.	The health and quality of tea in Anhui Province and its countermeasures	Journal of Tea Business	2004
107.	Preliminary analysis on chemical composition of Baojing Golden Tea	Tea Communication	2008
108.	Determination of trace elements in tea by wavelength dispersive X-ray fluorescence spectroscopy	Nuclear Techniques	2013
109.	Analysis of mineral elements and tea polyphenols and other active	Jiangsu Agricultural Sciences	2015

	ingredients in the tea leaves of different tea fruits intercropped		
110.	The content and enrichment characteristics of 9 kinds of mineral elements in different tea plant varieties	Jiangsu Agricultural Sciences	2019
111.	The principal component analysis of metal elements in green tea	Journal of Southwest University of Science and Technology	2011
112.	Extraction rate of trace lead in green tea with different soaking conditions	Chinese Journal of Food Hygiene	2014
113.	Effects of different mother material soils on biochemical characteristics of oolong tea	Journal of Fujian Agricultural University	1999
114.	Detection of heavy metal content in different varieties of chrysanthemum tea and analysis of dissolution rate	Chinese Journal of Health Inspection	2018
115.	Analysis of the chemical component on quality of Rougui tea in different rock areas	Journal of Food Safety and Quality	2015
116.	Determination of lead in tea by incomplete digestion-isotope dilution-inductively coupled plasma mass spectrometry	Journal of Food Safety and Quality	2018
117.	Investigation of lead and chromium in tea from some markets in China	Journal of Food Safety and Quality	2014
118.	Determining mainly heavy metal elements and rare earth elements in Ya' an Tibetan tea by ICP - MS	A Dissertation Submitted to Sichuan Agricultural University in Partial Fulfillment of the Requirements for the Professional Degree of Master of Agriculture Extension	2019
119.	Correlation analysis and evaluation of heavy metals in tea and in soil —A Case Study of Fanjiayuan Tea Farm in Miluo	Hunan Agricultural Sciences	2021
120.	Determination of the content of As and Hg in tea leaves and the influence of the soaking method on their leaching amount	Cantonese tea	2010
121.	The trage Elements in the tea	Jiangxi Chemical	2004
122.	Research on the contents detection of metal elements and its extracting characteristics in tea	Chinese Journal of Health Inspection	2016
123.	Study on Simultaneous Determination of Phosphorus, Potassium, Lead, Zinc etc. altogether 17 Elements in Tea	Food Science	2006
124.	Analysis and hazard evaluation of aluminium, lead, arsenic, mercury, chromium and cadmium content in tea	Journal of Food Safety and Quality	2021
125.	Lead chrome green determination in tea by inductive coupled plasma atomic emission spectrometry	Journal of Anshan Normal University	2013
126.	Determination method comparison of lead content in tea and Its leaching rule	Joumal of Jiamusi University (Natural Science Edition)	2010
127.	Determination of contents of two elements of Cu, Pb in tea samples	Journal of Kaili University	2007
128.	Detection and analysis on the microelement of Tea	Value Enigineering	2015
129.	Lixiviation of trace elements from Tea	The world of chemistry	2004
130.	Studies on techniques of rapid determining heavy metals in Tea-leaves	Food Science	2004
131.	Hygienic study of harmful elements As, Ba, Cd and Pb in tea	Chinese Journal of Food Hygiene	2001

132.	Determination of heavy metals in tea and exploring the law of solvent extraction	Food Research And Development	2015
133.	Study on the leaching law of heavy metals in tea leaves	Chemical analytical metrology	2007
134.	Study on Leaching Rate of Lead in Tea	Branding and standardization	2018
135.	Analysis of spatial heterogeneity of soil heavy metals in tea plantation: Case study of high quality tea garden in Jiangsu and Zhejiang	Geographical Research	2017
136.	Determination of lead in tea by plating mercury anodic stripping voltammetry	Science and Technology of Food Industry	2015
137.	A study on the contents of Iron, Zinc, Copper in common tea Leaves	Journal of Quanzhou Normal University	2017
138.	Investigation and analysis of lead and copper content in tea in Chengdu	Journal of Sichuan Continuing Education College of MS	1994
139.	Mineral element characteristics analysis and origin identification of Daishan Penglai Xianzhi green tea	China Tea	2016
140.	Simultaneous determination of 20 elements in tea by inductively coupled plasma-mass spectrometry	Journal of Food Safety and Quality	2018
141.	Determination of 14 kinds of elements in roselle by inductively coupled plasma mass spectrometry	Journal of Food Safety and Quality	2019
142.	Analysis of trace elements content in chrysanthemum teas and their dissolution characteristics by inductively coupled plasma-mass spectrometry	Journal of Food Safety and Quality	2016
143.	Determination of Trace Elements and Principal Component Analysis in Duyun Maojian Tea	Modern agricultural technology	2016
144.	Determination of lead, copper, arsenic, cadmium, chromium, and cobalt in tea by end-view ICP - AES method	Physical and Chemical Inspection (Chemistry Fascicle)	2003
145.	Fanjing mountains white tea content of inorganic elements analysis	Food Industry	2017
146.	Appraisal on environmental quality of Fanjing tea production base	Guizhou Agricultural Sciences	2010
147.	Principal component analysis, correlation and cluster analysis of 18 mineral elements in Fanjing mountains tea	Natural product research and development	2017
148.	Analysis of nutritional components of Fanjing mountain dark tea	Food Research And Development	2020
149.	Risk evaluation of heavy metal elements contamination on “Fenghuang Dancong”Oolong Tea	Food Science	2008
150.	Discussion on the current situation of copper content of tea in Fujian and the pollution of the initial production process	Fujian tea	2001
151.	Lead Content and Isotopic Tracing for Soil-tea System of Some Tea Gardens in Fujian Province, China	Earth and Environment	2018
152.	Fourteen kinds of elements in teas from Fujian province	Chinese Journal of Food Hygiene	2011
153.	Comparative analysis of biochemical components and trace metal elements of different varieties of tea in Longnan, Gansu Province	Packaging & Food Machinery	2019
154.	Determination of seven microelement in green tea of three regions in Gannan	Guangdong Weiliang Yuansu Kexue	2005
155.	Determination of metal elements in extracting traits of mountain	Journal of Anhui Agricultural	2011

	green tea	University	
156.	Determination of heavy metals in tea through coprecipitation separation and enrichment combined with partial least square − Spectrophotometry	Analysis and Testing Technology and Instruments	2016
157.	ICP - MS Analysis on mineral and heavy metal elements of Hakka roasted green tea in Dongyuan, Guangdong	Guangdong Agricultural Sciences	2016
158.	Analysis of trace element content and water dissolution of Guangdong Yingde black tea and green tea	Chinese Journal of Ethnic and Folk Medicine	2004
159.	Comparison of quality of Hawk tea (Litsea coreana var. sinensis) in four counties of Guizhou Province	Acta Agriculturae Zhejiangensis	2021
160.	The analysis of mineral element content of Guizhou tea and relation with the grade of tea	Trace Elements and Health Research	1998
161.	Heavy metal content and pollution evaluation in Guizhou tea	Agricultural Services	2020
162.	The chemical constituents of Laoying Tea from Guizhou	Journal of Plant Resources and Environment	2001
163.	Geographical origin traceability of green tea from Fanjing Mountain Area, Tongren, Guizhou	Food Science	2021
164.	Studies on trace elements in green tea in northern Guangxi	Journal of Youjiangf Medical College for National Minorities	1993
165.	A comparative analysis on dissolvable rate of trace elements from Hainan Kudingchaand green tea	Studies of Trace Elements and Health	2008
166.	Pollution characteristics and health risk assessment of nine elements in commercially available Kuding and Partridge tea leaves and their infusions in Hainan Province	Practical preventive medicine	2021
167.	Determination of extract and trace element content of Hanzhong green tea water	Journal of Tea Business	2008
168.	Investigation of nutrient and heavy metals in tea garden soil in Hangzhou	Chinese Agricultural Science Bulletin	2017
169.	Study on the dissolution rate of trace elements of 8 kinds of tea in Honghe State	Henan Agricultural Sciences	2007
170.	Studies on mineral elements content in the famous green tea of Hubei	Hubei Agricultural Sciences	2010
171.	The study of the contents of lead in green teas processed by two types of energy in Yichang，Hubei Province	Guangdong Weiliang Yuansu Kexue	2015
172.	Analysis of ingredient and the appraisal of security of Hunan Tiancha	Journal of Hunan Agricultural University (Natural Sciences)	2004
173.	Huzhou city sells substitute tea and green tea metal elements and rare earth elements test results	Preventive medicine	2019
174.	Current status and health risk assessment of metal contamination in commercial tea in Huizhou City in 2019	Chinese Journal of Health Inspection	2021
175.	Detection of lead, cadmium and chromium in instant tea by mixed matrix modifier-graphite furnace atomic absorption spectrometry	Journal of Food Safety and Quality	2015
176.	Study on determination of five trace elements in teas by flame	Guangdong Weiliang Yuansu Kexue	2004

	atomization method		
177.	Flame atomic absorption spectroscopy for the determination of trace elements in Shaanxi green tea and black tea	Chemistry & Bioengineering	2020
178.	Determination of trace elements in tea by FAAS and FAFS	Chinese Journal of Spectroscopy Laboratory	2010
179.	Determination of Cr（III）and Cr（VI）in Tea by IC－ICP－MS based on ASE Extraction	Hubei Agricultural Sciences	2017
180.	Green tea differentiation using multistatistical analysis based on content of mineral elements	Journal of Liaoning Shihua University	2013
181.	Identification of tea based on mineral content and support vector machines	Journal of Jiangsu University (Natural Science Edition)	2011
182.	Longjing tea origin traceability study based on biochemical composition and mineral elements	Zhejiang Agricultural Sciences	2017
183.	Several different digestion methods and detection methods compare of determine lead content of green tea	Metrology and testing technology	2013
184.	The content of cu in several kinds of tea	Journal of Yunnan University	2013
185.	Research on the content of pb and cd in some teas	Journal of Chongqing Technology and Business University (Natural Science Edition)	2011
186.	Analysis of pollution of heavy metal elements of several green tea in Guizhou	Science and Technology of Food Industry	2011
187.	Determination of mercury in several commercially available green tea	Shandong Chemical Industry	2020
188.	Multivariate analysis of heavy metals in fresh tea leaves from Jiangxi Province	Journal of Food and Biotechnology	2012
189.	Assessment discussion on the degree of heavy metals in fresh tea leaves from Jiangxi Province	Food and Frementation Industries	2011
190.	Determination of ten kind of metal elements in different varieties of Jiangxi famous tea	Food & Machinery	2020
191.	Nutritional analysis and evaluation of chrysanthemum	Modern Food Science and Technology	2019
192.	Analysis on dissolving of metal elements in Yunnan Fengqing Tea by Immersion Time	Journal of Kunming University	2016
193.	Effects of immersion temperatures on dissolving of metal elements in different tea infusions	Journal of Kunming University	2017
194.	Investigation and analysis of lead residual in commercially available Pu'er tea in Kunming	Chinese Journal of Health Inspection	2018
195.	Eagle tea ingredient research	Shi Zhen Chinese medicine Chinese medicine	2006
196.	Migration and accumulation of heavy metals in Hawk Tea	Journal of Nuclear Agricultural Sciences	2016
197.	Analysis of nutrient composition of Quinoa Seedlings and Development of quinoa green tea	Professional master's degree thesis of Jilin Agricultural University	2018
198.	Trace element determination in teas and discrimination analysis for	Rock and Mineral Analysis	2006

	Teas		
199.	Lingyun pekoe tea is mainly a mineral element with the relationship between tea quality	Guangxi hot work technology	1997
200.	Determination of 10 kinds of elements in Liubao Tea by inductively coupled plasma mass spectrometry	Shangdong Chemical Industry	2019
201.	A comparative study on the dissolution characteristics of six kinds of domestic black tea and Sri Lanka Black Tea	Journal of Green Science and Technology	2017
202.	Influence factors on lead contents in Longjing Tea	.Journal of Agro-Entironment Science	2004
203.	Chemical constituent's difference analysis of green tea or black tea or white tea and oolong	Forestry Science	2011
204.	Determination of heavy metal content in green tea and extracts	Journal of Beijing Technology and Business University(Natural Science Edition)	2012
205.	Study on contents of primary speciation of trace elements in green tea and red tea	Chinese Journal of Spectroscopy Laboratory	2004
206.	Analysis and distribution characteristics of 16 elements in green tea	Chinese Journal of Health Engineering	2019
207.	Content characteristics of mineral elements and its correlation with soil nutrients and effective components in Chrysanthemum morifolium from different producing areas in Macheng	China Journal of Chinese Materia Medical	2021
208.	Analysis of main chemical components of mengding yellow bud	Food Science	2014
209.	Analysis and evaluation of nutritional components of aglossa dimidiata-Litsea coreana insect tea	Guangdong Agricultural Sciences	2013
210.	Analysis of 23 mineral elements in tea samples collected from china and japan by using ICP -AES and ICP -MS combined with a closed decomposition	Spectroscopy and Spectral Analysis	2005
211.	Appraisal on envirormental quality of Fanjing tea production base	Guizhou Agricultural Sciences	2010
212.	The Problem of Lead Contamination and Its Source in Tea Leaves	Guandong Weiliang Yuansu Kexue	2004
213.	Research progress on the current situation of lead content in tea and its control technology	China Tea	2008
214.	Analysis of spatial heterogeneity of soil heavy metals in tea garden based on township scale—a case study of high-quality famous tea planting area in Jiangsu and Zhejiang	A Dissertation Submitted in Partial Fulfillment of the Requirements for the Degree of Master of Science	2018
215.	Comparison of the quality and agricultural geological traits of "Umeng morning" tea in southern Sichuan	Jiangsu Agricultural Sciences	2015
216.	The evalution for heavy metal content in the tea garden soil in west of Sichuan	Journal of Agro - Environment Science	2005
217.	Analysis of the spatial variation and evaluation on soil fertility and heavy metal of typical green tea plantations	Chinese Academy of Agricultural Sciences Dissertation	2015
218.	Evaluation of soil environment quality in the production regions of famous green tea in Fujian	Journal of Fujian Agricultural University	1998
219.	Distribution and migration characteristics of cadmium and lead in soil-tea tree system	Journal of Fujian Agriculture and Forestry University(Natural Science Edition)	2019

220.	Environmental quality evaluation of pollution − free Jasmine scented tea plantations in Hengxian, Guangxi	Guangxi Agricultural Sciences	2009
221.	Vertical variation of heavy metals in soil of Guiding tea areas	Guangdong Agricultural Sciences	2014
222.	Geochemical characteristics of cultivated land in Yuqing Country, Guizhou Province and its influence on the quality of rice, tea and other agricultural products	Mineral Exploration	2020
223.	Evaluation of Heavy Metal Pollution of Tea Garden in Hubei	Gansu Agricultural Science and Technology	2017
224.	Soil heavy metal pollution status and evaluation of typical tea gardens in Hubei Province	China Tea	2009
225.	Characteristics of tea plantation soil environmental. Quality around the Dongting Lake in Hunan province	Master's degree thesis of Hunan Agricultural University	2007
226.	Soil quality and safety investigation and evaluation of tea gardens in Guiding County based on GIS technology	Jiangsu Agricultural Sciences	2014
227.	Soil heavy metal content and safety and quality evaluation of tea gardens in Laoshan District	Shangdong Agricultural Sciences	2009

Table S2 Average daily exposure reference dose and cancer slope factor of various heavy metals

Elements	RfD (mg/(kg·d))[1]	SF (mg/(kg·d)) [2]
As	0.0003	1.5
Cd	0.0010	15
Cr	0.0030	0.5
Cu	0.0400	
Hg	0.0003	
Pb	0.0035	0.0085

Table S3 Concentration of heavy metals and pollution rates in tea and tea plantation soils

Elements	Tea samples			Tea plantation soil samples			
	Range (mg/kg)	Mean (mg/kg)	Pollution rate (%)	Range (mg/kg)	Mean (mg/kg)	Pollution rates 1(%)	Pollution rates 2 (%)
As	0–5.64	0.21	0.21	0.1–75.86	12.46	0.44	
Cd	0–3.79	0.14	1.18	0–1.29	0.16	6.38	99.80
Cr	0.01–42.94	1.17	2.10	0.3–254.4	58.31	0.95	100
Cu	0–132.31	14.6	2.87	1.53–113.6	23.36	1.73	
Hg	0–1.81	0.04	1.56	0.01–0.42	0.17	12.33	90.40
Pb	0–31.73	1.09	1.04	0.04–157.6	40.36	0	99.75

Pollution rate 1 is calculated according to Chinese standards. Pollution rate 2 is calculated according to WHO standards.

Table S4 Heavy metal concentration and pollution ratio in different kinds of tea

		Green Tea	White Tea	Dark Tea	Black Tea	Oolong Tea	Scented Tea	Yellow Tea
As	Mean (mg/kg)	0.23	0.31	0.18	0.11	0.14	0.34	0.05
	sample size	2732	50	716	608	343	334	20
	Pollution ratio(%)	0	0	0	0	0	2.99	0
Cd	Mean (mg/kg)	0.16	0.13	0.08	0.07	0.12	0.21	0.37
	sample size	3121	62	679	602	369	404	20
	Pollution ratio(%)	0.64	0	1.33	1.33	2.17	4.21	0
Cr	Mean (mg/kg)	1.08	0.69	1.77	0.61	0.70	3.61	0.39
	sample size	2832	49	750	616	449	192	15
	Pollution ratio(%)	0.32	0	6.30	0.65	4.01	13.0	0
Cu	Mean (mg/kg)	14.9	14.2	14.9	16.3	13.3	13.4	14.4
	sample size	2810	54	657	467	527	342	24
	Pollution ratio(%)	3.17	3.70	0.46	6.42	1.52	1.75	8.33
Hg	Mean (mg/kg)	0.04	0.09	0.03	0.03	0.04	0.06	0.30
	sample size	2465	41	596	465	171	281	7
	Pollution ratio(%)	1.95	0	0	0	0	5.34	0
Pb	Mean (mg/kg)	1.17	0.66	1.10	0.70	0.81	1.57	0.84
	sample size	3868	63	846	697	669	449	19
	Pollution ratio(%)	0.59	0	3.19	0.29	0.15	3.56	0
Sum pollution ratio(%)		6.67	3.70	11.24	8.69	7.84	30.88	8.33

Table S5 The non-carcinogenic risk assessment of tea consumption for male adults in China

Region	HQ of As	HQ of Cd	HQ of Cr	HQ of Cu	HQ of Hg	HQ of Pb	HI
Heilongjiang	0.0006	0.0002	0.0003	0.0020	0.0001	0.0007	0.0039
Xinjiang	0.0110	0.0049	0.0015	0.0080	0.0004	0.0033	0.0291
Shanxi	0.0056	0.0022	0.0015	0.0049	0.0012	0.0041	0.0195
Ningxia	0.0030	0.0010	0.0011	0.0080	0.0007	0.0031	0.0169
Tibet	0.0302	0.0097	0.0105	0.0816	0.0070	0.0308	0.1698
Shandong	0.0270	0.0025	0.0027	0.0123	0.0145	0.0119	0.0710
Henan	0.0074	0.0035	0.0020	0.0047	0.0027	0.0043	0.0246
Jiangsu	0.0060	0.0027	0.0014	0.0040	0.0018	0.0065	0.0223
Anhui	0.0203	0.0090	0.0036	0.0130	0.0065	0.0117	0.0642
Hubei	0.0071	0.0016	0.0010	0.0038	0.0020	0.0032	0.0187
Zhejiang	0.0048	0.0011	0.0016	0.0054	0.0009	0.0054	0.0193
Jiangxi	0.0037	0.0018	0.0011	0.0039	0.0016	0.0024	0.0144
Hunan	0.0039	0.0035	0.0009	0.0042	0.0012	0.0035	0.0172
Yunnan	0.0192	0.0096	0.0122	0.0163	0.0092	0.0113	0.0777
Guizhou	0.0145	0.0071	0.0042	0.0059	0.0065	0.0081	0.0463
Fujian	0.0083	0.0023	0.0016	0.0071	0.0039	0.0062	0.0292
Guangxi	0.0028	0.0179	0.0008	0.0037	0.0009	0.0024	0.0285
Guangdong	0.0103	0.0095	0.0035	0.0151	0.0128	0.0141	0.0653
Hainan	0.0020	0.0142	0.0006	0.0014	0.0002	0.0018	0.0202
Jilin	0.0012	0.0005	0.0008	0.0047	0.0004	0.0015	0.0092
Liaoning	0.0050	0.0015	0.0012	0.0099	0.0016	0.0033	0.0227
Tianjin	0.0037	0.0014	0.0006	0.0055	0.0019	0.0032	0.0162
Qinghai	0.0037	0.0012	0.0013	0.0098	0.0009	0.0038	0.0207
Gansu	0.0197	0.0019	0.0055	0.0109	0.0080	0.0095	0.0555
Shaanxi	0.0222	0.0033	0.0037	0.0062	0.0154	0.0079	0.0586
Inner Mongolia	0.0025	0.0007	0.0013	0.0074	0.0009	0.0019	0.0147
Chongqing	0.0174	0.0052	0.0031	0.0084	0.0046	0.0071	0.0459
Hebei	0.0091	0.0027	0.0011	0.0087	0.0039	0.0063	0.0319
Shanghai	0.0022	0.0010	0.0009	0.0049	0.0007	0.0044	0.0141
Beijing	0.0078	0.0023	0.0010	0.0102	0.0022	0.0134	0.0370
Taiwan	0.0023	0.0009	0.0084	0.0032	0.0003	0.0057	0.0207
Hong Kong	0.0055	0.0015	0.0016	0.0109	0.0055	0.0058	0.0308
Macau	0.0000	0.0000	0.0000	0.0000	0.0000	0.0000	0.0000
Sichuan	0.0122	0.0025	0.0019	0.0074	0.0024	0.0109	0.0372
Average	0.0089	0.0039	0.0025	0.0092	0.0036	0.0065	0.0345

Table S6 The non-carcinogenic risk assessment of tea consumption for female adults in China

Region	HQ of As	HQ of Cd	HQ of Cr	HQ of Cu	HQ of Hg	HQ of Pb	HI
Heilongjiang	0.0007	0.0003	0.0003	0.0023	0.0002	0.0009	0.0045
Xinjiang	0.0127	0.0057	0.0018	0.0092	0.0004	0.0038	0.0336
Shanxi	0.0064	0.0026	0.0017	0.0057	0.0014	0.0048	0.0226
Ningxia	0.0035	0.0011	0.0012	0.0093	0.0008	0.0036	0.0195
Tibet	0.0348	0.0112	0.0122	0.0943	0.0081	0.0355	0.1962
Shandong	0.0312	0.0029	0.0031	0.0142	0.0168	0.0138	0.0820
Henan	0.0086	0.0040	0.0024	0.0054	0.0031	0.0050	0.0284
Jiangsu	0.0069	0.0031	0.0017	0.0046	0.0020	0.0075	0.0258
Anhui	0.0234	0.0104	0.0042	0.0150	0.0076	0.0135	0.0741
Hubei	0.0082	0.0019	0.0011	0.0043	0.0024	0.0037	0.0216
Zhejiang	0.0055	0.0013	0.0019	0.0063	0.0011	0.0063	0.0222
Jiangxi	0.0043	0.0021	0.0012	0.0045	0.0019	0.0027	0.0167
Hunan	0.0045	0.0040	0.0010	0.0049	0.0014	0.0040	0.0199
Yunnan	0.0222	0.0110	0.0140	0.0188	0.0106	0.0131	0.0897
Guizhou	0.0168	0.0082	0.0049	0.0068	0.0075	0.0093	0.0535
Fujian	0.0096	0.0026	0.0018	0.0081	0.0045	0.0072	0.0337
Guangxi	0.0033	0.0207	0.0009	0.0043	0.0011	0.0028	0.0330
Guangdong	0.0119	0.0110	0.0041	0.0174	0.0148	0.0163	0.0755
Hainan	0.0023	0.0165	0.0006	0.0016	0.0002	0.0021	0.0233
Jilin	0.0014	0.0006	0.0010	0.0055	0.0004	0.0017	0.0106
Liaoning	0.0058	0.0018	0.0014	0.0114	0.0018	0.0039	0.0262
Tianjin	0.0043	0.0016	0.0006	0.0063	0.0022	0.0037	0.0187
Qinghai	0.0043	0.0014	0.0015	0.0113	0.0010	0.0044	0.0240
Gansu	0.0227	0.0022	0.0064	0.0126	0.0092	0.0110	0.0641
Shaanxi	0.0257	0.0038	0.0043	0.0071	0.0177	0.0091	0.0677
Inner Mongolia	0.0029	0.0009	0.0015	0.0085	0.0011	0.0021	0.0169
Chongqing	0.0201	0.0060	0.0036	0.0098	0.0054	0.0082	0.0530
Hebei	0.0105	0.0032	0.0013	0.0101	0.0045	0.0073	0.0369
Shanghai	0.0025	0.0011	0.0011	0.0056	0.0008	0.0051	0.0162
Beijing	0.0091	0.0027	0.0011	0.0118	0.0026	0.0155	0.0427
Taiwan	0.0026	0.0010	0.0097	0.0037	0.0003	0.0066	0.0239
Hong Kong	0.0063	0.0017	0.0018	0.0126	0.0064	0.0067	0.0355
Macau	0.0000	0.0000	0.0000	0.0000	0.0000	0.0000	0.0000
Sichuan	0.0141	0.0029	0.0022	0.0085	0.0028	0.0126	0.0430
Average	0.0103	0.0045	0.0029	0.0106	0.0042	0.0075	0.0399

Table S7 The carcinogenic risk assessment of tea consumption for male adults in China

Region	Risk of As	Risk of Cd	Risk of Cr	Risk of Pb	Total Risk
Heilongjiang	2.61E-07	3.36E-06	3.78E-07	2.23E-08	4.02E-06
Xinjiang	4.95E-06	7.40E-05	2.32E-06	9.69E-08	8.13E-05
Shanxi	2.51E-06	3.32E-05	2.21E-06	1.23E-07	3.81E-05
Ningxia	1.36E-06	1.46E-05	1.59E-06	9.18E-08	1.77E-05
Tibet	1.36E-05	1.46E-04	1.58E-05	9.15E-07	1.76E-04
Shandong	1.22E-05	3.72E-05	4.04E-06	3.55E-07	5.38E-05
Henan	3.33E-06	5.18E-05	3.07E-06	1.29E-07	5.83E-05
Jiangsu	2.69E-06	4.01E-05	2.16E-06	1.94E-07	4.51E-05
Anhui	9.13E-06	1.35E-04	5.42E-06	3.49E-07	1.50E-04
Hubei	3.20E-06	2.46E-05	1.47E-06	9.50E-08	2.94E-05
Zhejiang	2.14E-06	1.68E-05	2.43E-06	1.61E-07	2.16E-05
Jiangxi	1.66E-06	2.72E-05	1.60E-06	7.03E-08	3.05E-05
Hunan	1.75E-06	5.24E-05	1.32E-06	1.04E-07	5.56E-05
Yunnan	8.65E-06	1.43E-04	1.82E-05	3.36E-07	1.71E-04
Guizhou	6.53E-06	1.06E-04	6.37E-06	2.40E-07	1.20E-04
Fujian	3.72E-06	3.38E-05	2.34E-06	1.84E-07	4.01E-05
Guangxi	1.28E-06	2.68E-04	1.20E-06	7.14E-08	2.71E-04
Guangdong	4.62E-06	1.43E-04	5.29E-06	4.20E-07	1.54E-04
Hainan	8.83E-07	2.14E-04	8.31E-07	5.44E-08	2.15E-04
Jilin	5.53E-07	7.27E-06	1.26E-06	4.39E-08	9.12E-06
Liaoning	2.27E-06	2.31E-05	1.86E-06	9.95E-08	2.74E-05
Tianjin	1.67E-06	2.12E-05	8.31E-07	9.41E-08	2.38E-05
Qinghai	1.69E-06	1.81E-05	1.96E-06	1.14E-07	2.19E-05
Gansu	8.86E-06	2.87E-05	8.26E-06	2.84E-07	4.61E-05
Shaanxi	9.99E-06	4.92E-05	5.58E-06	2.34E-07	6.50E-05
Inner Mongolia	1.11E-06	1.12E-05	1.95E-06	5.51E-08	1.43E-05
Chongqing	7.84E-06	7.76E-05	4.66E-06	2.12E-07	9.03E-05
Hebei	4.11E-06	4.11E-05	1.63E-06	1.89E-07	4.70E-05
Shanghai	9.88E-07	1.44E-05	1.41E-06	1.32E-07	1.69E-05
Beijing	3.53E-06	3.52E-05	1.46E-06	4.00E-07	4.06E-05
Taiwan	1.02E-06	1.30E-05	1.26E-05	1.70E-07	2.67E-05
Hong Kong	2.47E-06	2.27E-05	2.35E-06	1.72E-07	2.77E-05
Macau	0.00E+00	0.00E+00	0.00E+00	0.00E+00	0.00E+00
Sichuan	5.48E-06	3.75E-05	2.86E-06	3.23E-07	4.61E-05
Average	4.00E-06	5.78E-05	3.73E-06	1.92E-07	6.57E-05

Table S8 The carcinogenic risk assessment of tea consumption for female adults in China

Region	Risk of As	Risk of Cd	Risk of Cr	Risk of Pb	Total Risk
Heilongjiang	3.01E-07	3.88E-06	4.37E-07	2.58E-08	4.64E-06
Xinjiang	5.72E-06	8.55E-05	2.68E-06	1.12E-07	9.40E-05
Shanxi	2.90E-06	3.84E-05	2.56E-06	1.42E-07	4.40E-05
Ningxia	1.57E-06	1.69E-05	1.83E-06	1.06E-07	2.04E-05
Tibet	1.57E-05	1.68E-04	1.83E-05	1.06E-06	2.04E-04
Shandong	1.40E-05	4.30E-05	4.66E-06	4.11E-07	6.21E-05
Henan	3.85E-06	5.98E-05	3.55E-06	1.49E-07	6.74E-05
Jiangsu	3.11E-06	4.63E-05	2.49E-06	2.24E-07	5.22E-05
Anhui	1.05E-05	1.56E-04	6.26E-06	4.03E-07	1.73E-04
Hubei	3.70E-06	2.84E-05	1.70E-06	1.10E-07	3.39E-05
Zhejiang	2.47E-06	1.95E-05	2.81E-06	1.86E-07	2.49E-05
Jiangxi	1.91E-06	3.14E-05	1.85E-06	8.13E-08	3.52E-05
Hunan	2.02E-06	6.06E-05	1.52E-06	1.20E-07	6.42E-05
Yunnan	1.00E-05	1.66E-04	2.11E-05	3.88E-07	1.97E-04
Guizhou	7.54E-06	1.23E-04	7.35E-06	2.77E-07	1.38E-04
Fujian	4.30E-06	3.91E-05	2.71E-06	2.13E-07	4.63E-05
Guangxi	1.47E-06	3.10E-04	1.38E-06	8.25E-08	3.13E-04
Guangdong	5.34E-06	1.65E-04	6.11E-06	4.85E-07	1.77E-04
Hainan	1.02E-06	2.47E-04	9.60E-07	6.28E-08	2.49E-04
Jilin	6.39E-07	8.39E-06	1.45E-06	5.07E-08	1.05E-05
Liaoning	2.62E-06	2.67E-05	2.15E-06	1.15E-07	3.16E-05
Tianjin	1.92E-06	2.45E-05	9.60E-07	1.09E-07	2.75E-05
Qinghai	1.95E-06	2.09E-05	2.27E-06	1.31E-07	2.53E-05
Gansu	1.02E-05	3.32E-05	9.54E-06	3.28E-07	5.33E-05
Shaanxi	1.15E-05	5.68E-05	6.45E-06	2.71E-07	7.51E-05
Inner Mongolia	1.28E-06	1.29E-05	2.26E-06	6.37E-08	1.65E-05
Chongqing	9.06E-06	8.96E-05	5.38E-06	2.45E-07	1.04E-04
Hebei	4.75E-06	4.75E-05	1.88E-06	2.18E-07	5.43E-05
Shanghai	1.14E-06	1.66E-05	1.63E-06	1.52E-07	1.95E-05
Beijing	4.07E-06	4.06E-05	1.69E-06	4.62E-07	4.69E-05
Taiwan	1.18E-06	1.50E-05	1.45E-05	1.96E-07	3.09E-05
Hong Kong	2.85E-06	2.62E-05	2.71E-06	1.99E-07	3.20E-05
Macau	0.00E+00	0.00E+00	0.00E+00	0.00E+00	0.00E+00
Sichuan	6.34E-06	4.33E-05	3.31E-06	3.73E-07	5.33E-05
Average	4.62E-06	6.68E-05	4.31E-06	2.22E-07	7.59E-05

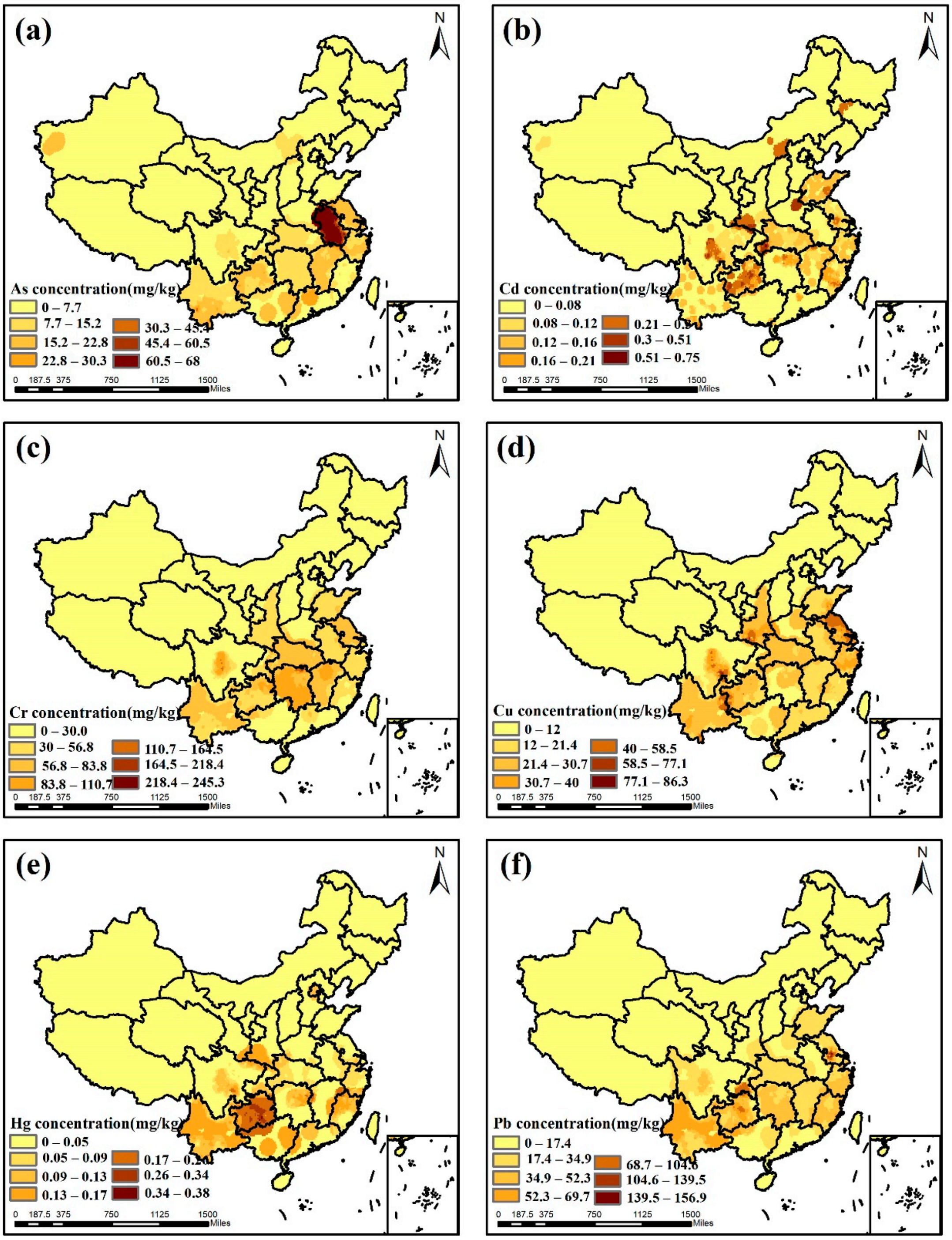

Figure S1. Spatial distribution of heavy metals concentrations in tea plantation soils. (a): As; (b): Cd; (c): Cr; (d): Cu; (e): Hg; (f): Pb.

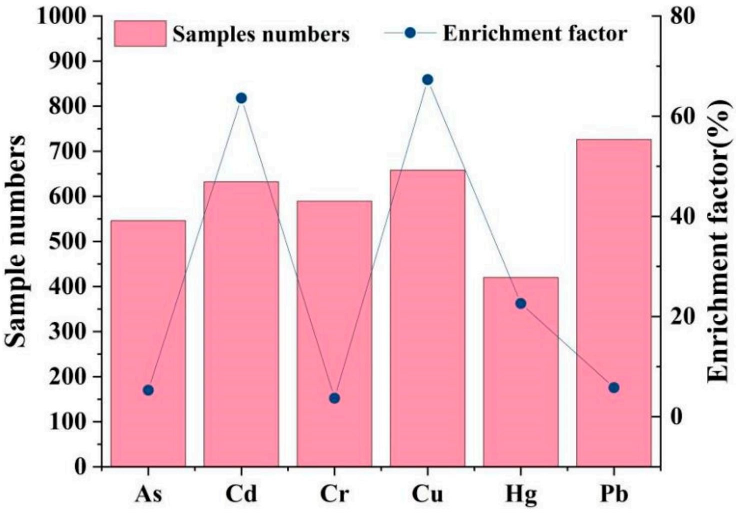

Figure S2. The number of samples from the same location used for different heavy metals and the calculated EF averages.

References

1. USEPA. Regional Screening Level (RSL) Summary Table, Washington, DC, USA, 2015.

2.Zhang, L.e.; Mo, Z.; Qin, J.; Li, Q.; Wei, Y.; Ma, S.; Xiong, Y.; Liang, G.; Qing, L.; Chen, Z. et al. Change of water sources reduces health risks from heavy metals via ingestion of water, soil, and rice in a riverine area, South China. *Sci. Total. Environ*. **2015**, 530-531,163-170.
